# Gene expression in Verson’s glands of the fall armyworm suggests their role in molting and immunity

**DOI:** 10.3389/finsc.2023.1124278

**Published:** 2023-02-10

**Authors:** Jinmo Koo, Xien Chen, Subba Reddy Palli

**Affiliations:** Department of Entomology, College of Agriculture, University of Kentucky, Lexington, KY, United States

**Keywords:** *Spodoptera frugiperda*, transcriptome, molting fluid, cuticular protein, secretion, antimicrobial peptides

## Abstract

Verson’s glands are segmental pairs of dermal glands attached to the epidermis in lepidopteran larvae. They produce macromolecules during intermolt period and empty them during each molt. Morphological, histochemical, developmental, and protein analysis studies have been conducted to determine the functions of Verson’s glands. However, the exact role of Verson’s glands remains unclear. In our previous study, a strain of transgenic fall armyworm, *Spdoptera frugiperda* expressing green fluorescence protein (GFP) and Systemic RNA interference defective protein 1 (SID1) from *Caenorhabditis elegans* was established to improve RNA interference (RNAi) efficiency. Unexpectedly, we found that GFP fluorescence was significantly brighter in Verson’s glands than in other tissues. Also, RNAi efficiency improved more in Verson’s glands than in other tissues. We took advantage of improved RNAi efficiency to explore the function of Verson’s glands. RNA-seq analysis revealed that genes highly expressed in Verson’s glands code for cuticular proteins, molting fluid proteins, hemolymph proteins, and antimicrobial peptides. Injection of dsRNA targeting essential genes, inhibitor of apoptosis (IAP), Actin, and vacuolar-type ATPase (VATPase) interfered with Verson’s glands growth. These results revealed that Verson’s glands may contribute to hemolymph, cuticle, molting fluid, and immune response during molting. This study also provide useful tools for future research in identifying the physiological role of Verson’s glands in lepidopteran insects.

## 1 Introduction

Verson’s glands (1) are derivatives of dermal cells in the epidermis (2). They occur in pairs located dorsally on each segment, ventrally on each leg, and anal prolegs (3). Verson’s glands contain three cells, a secretory cell, a saccule cell, and a duct cell (4, 5). The secretoty cell takes most of the volume of the Verson’s glands. It produce segment-specific proteins during larval and pupal molts (6). The size of the Verson’s glands correlates with the cycle of larval molt; they grow until molt and shrink after each molt. They reach the maximum size (50-fold increase) during the prepupal stage (7) and undergo programmed cell death after the larval-pupal molt. Synthesis of pupal-specific proteins and growth of Verson’s glands are regulated by juvenile hormones and ecdysteroids (8). The growth of Verson’s glands and the molting cycle are closely aligned, suggesting that Verson’s gland secretions contribute to molting fluid (9). Histochemical studies showed that the acidic sulfated mucopolysaccharide cement layer is secreted by Verson’s glands (4, 10). The fluid content of Verson’s glands has phenoloxidase activity (7). Such activity is also found in molting fluids and contributes to cuticle tanning and immunity. Immune proteins were identified in these glands confirming their antimicrobial function (11). Further studies showed that Verson’s glands secrete these immune proteins into the hemolymph (12). Together, morphological, histochemical, developmental, and proteomics studies implicated Verson’s glands in molting and antimicrobial defense. However, the exact function of Verson’s glands is still not known.

In our previous studies (13), a transgenic *Spodoptera frugiperda* (fall armyworm, FAW) strain expressing green fluorescence protein (GFP) and Systemic RNA interference defective protein 1 (SID1) from *Caenorhabditis elegans* under the control of hr5-IE1 and IE1 promoters, respectively was established (hereinafter referred to as FAW-SID1) to increase the RNA interference (RNAi) efficiency. IE1 promoter drives strong and ubiquitous expression in most insect tissues during all developmental stages (14, 15). Unexpectedly, we found that GFP fluorescence was significantly higher in Verson’s glands compared to that in other tissues. Also, RNAi efficiency was improved more in Verson’s glands compared to other tissues. Distinct GFP fluorescence makes it easier for Verson’s glands to be visualized and dissected, and efficient RNAi response enables gene knockdown studies. RNA-seq analysis was performed to identify genes highly expressed in Verson’s glands. By analyzing the function of the highly expressed genes, we confirmed previous reports on Verson’s glands function in molting and immune response.

## 2 Materials and methods

### 2.1 Transgenic FAW establishment and insect rearing

A transgenic FAW-SID1 line was established as described previously (13). Insects were maintained at 27 ± 1°C, 60 ± 10% RH. Larvae were reared on artificial diet purchased from Southland Product Inc. (Arkansas, USA). Adults were fed on 10% sucrose solution.

### 2.2 Imaging

Nikon SMZ745 stereo microscope (Nikon, Japan) was used to take images. For taking images of tissues mounted on a slide, Olympus IX71 inverted microscope (Olympus Life Science, Japan) was used.

### 2.3 RNA-seq analysis

Verson’s glands were dissected from three different developmental stages of FAW-SID1 (L6 last instar larva, prepupa, and pupa), and total RNA was isolated using Trizol (Molecular Research Center Inc., Cincinnati, OH). The quantity and purity of isolated RNA were determined using the Nanodrop 2000 spectrophotometer (Thermo Fisher Scientific, USA). RNA-seq was done at Beijing Genomics Institute. The quality of RNA samples was checked, and the libraries were prepared and sequenced using BGISEQ-500 NGS platform. The sequences were assembled and analyzed in CLC Genomic Workbench (Version 9.5.9, Qiagen Bioinformatics, Valencia, CA). Raw RNA-seq data were mapped against FAW genome (16), with mapping options; mismatch cost 2, insertion cost 3, deletion cost 3, length fraction 0.8, and similarity fraction 0.8. Transcripts per million (TPM) was calculated to determine the expression levels. To find genes expressed higher in Verson’s glands compared to other tissues, sequence data of FAW fat body, midgut, and epidermis tissues from L6 last instar larva (17) were used. All three samples from different developmental stages (L6 last instar larva, prepupa, and pupa) of Verson’s glands were grouped together as replicates to compare with fat body, midgut, and epidermis samples, respectively. Upregulated genes in Verson’s glands compared to other tissues with FDR adjusted p-value (padj) < 0.01 were selected for further analysis. Gene Ontology identification of identified genes was performed using eggNOG-mapper (18). Gene Ontology enrichment analysis was done in the clusterprofiler package in R using ‘enricher’ function. For KEGG pathway enrichment analysis, the same package was used with ‘enrichKEGG’ function. FAW genes were blast searched in with *Drosophila melanogaster* database and *D. melanogaster* orthologs were used as input for KEGG pathway enrichment analysis. TPM of selected genes was used to generate heatmap using Morpheus (https://software.broadinstitute.org/morpheus/). Venn diagram was made using BioVenn (https://www.biovenn.nl/index.php). The RNA-seq raw data is deposited at GEO repository under the GEO accession number of GSE220983.

### 2.4 dsRNA injection

Total RNA isolated from FAW-SID1 Verson’s glands was used to synthesize cDNA, using M-MLV reverse transcriptase (Invitrogen™). 500 ng of total RNA was mixed with dNTP, random primer, and oligo primer in 13 µl volume. The mixture was incubated at 65°C for 5 min. Then 5X buffer, DTT, and reverse transcriptase were added to make up the total volume to 20 µl. The mixture was incubated at 37°C for 1 hour, followed by 70°C for 15 minutes for enzyme inactivation. Primers (**Table S1**) were designed to amplify fragments of target genes. PCR amplification was conducted in 50 µl reactions containing 5 µM each primer, 25 µl of 2x Taq master mix (NEB) and 2 µl cDNA. PCR conditions used were 95°C for 3 min, followed by 35 cycles of 95°C for 30s, 57°C for 30s and 68°C for 1 min, finishing with an extension step at 68°C for 5 min. PCR products were purified using the QIAquick PCR purification kit (QIAGEN). The purified PCR products were used as templates to synthesize dsRNAs using Megascript T7 RNA synthesis kit (Life Technologies, Carlsbad, CA). Using this method, dsRNA targeting the luciferase (Luc), GFP, inhibitor of apoptosis (IAP), actin, and vacuolar-type ATPase (VATPase) were synthesized. Before injection, FAW-SID1 larvae were immobilized by placing them on the ice for 10 minutes. Two µl containing 4 µg/µl dsRNA was injected into lateral side of abdominal segments of each larva. For injection, aspirator tube assembly fitted with a glass capillary needle was used. To make the glass capillary needle, 3.5” glass capillary tube was pulled by a needle puller (Model P-1000, Sutter Instrument, Novato, CA). After dsRNA injection, larvae were placed on ice for 20 minutes to prevent movement which could lead to leaking out of injected solution. Injected larvae were maintained in individual cups containing artificial diet.

### 2.5 Statistical analysis

To compare the means between two groups (control and treatment), a two-tailed t-test was used. To verify normal distribution of the replicates, Shapiro-Wilk test was performed using a tool from Statistics Kingdom website (https://www.statskingdom.com/shapiro-wilk-test-calculator.html). F-test with p value of 0.05 was used to determine equal variance among the treatment. t-test and F-test was done in Excel Version 2211 (Microsoft).

## 3 Results

### 3.1 Verson’s glands visualized by GFP fluorescence

**Figure 1:**
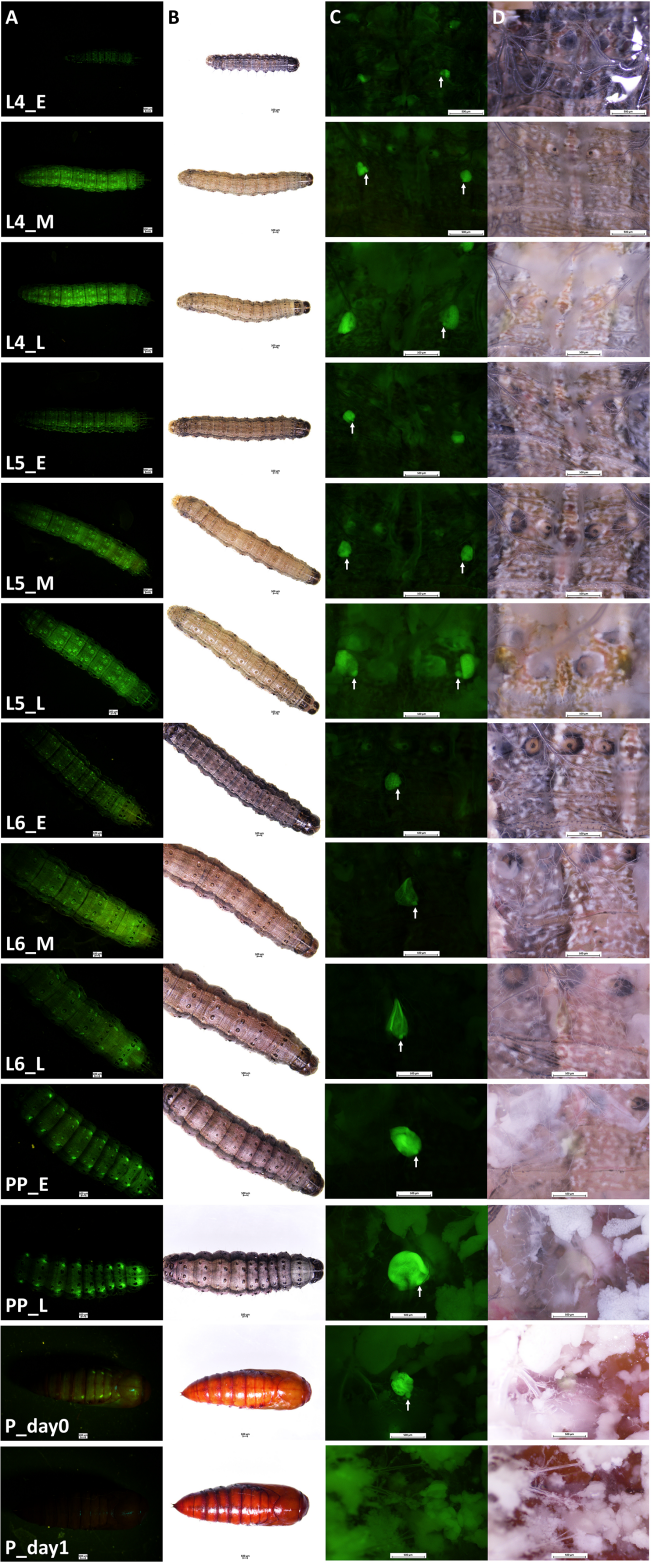
Verson’s glands visualized by GFP fluorescence. Verson’s glands from different developmental stages of FAW-SID1 insects are shown. **(A)**, GFP fluorescence image of a whole insect. **(B)**, bright field image of a whole insect. **(C)**, GFP fluorescence image of a dissected insect showing Verson’s glands in metathorax. **(D)**, bright field image of a dissected insect showing Verson’s glands in metathorax. L4, 4^th^ instar larva. L5, 5^th^ instar larva. L6, 6^th^ (last) instar larva. PP, prepupa. _E, early stage after molt. _M, middle stage. _L, late stage before molting except for the L6_L which indicates late L6 larval stage before entering wandering stage. P_day0, day 0 pupa. P_day1, day 1 pupa. White arrow indicates a saccule cell.

Transgenic FAW-SID1 showed high GFP fluorescence signal in Verson’s glands, which are visible even through the larval and pupal cuticle from outside (**Figure 1A**). Observation of Verson’s glands at different developmental stages (**Figure 1B**) revealed that Verson’s glands increased in size during the feeding period and decreased in size after molting. The size of Verson’s glands gradually increased as the larva developed and reached the maximum size by the end of larval period (**Figure 1C**). The size changes of the Verson’s glands were mainly accounted by a secretory cell, which takes most of the volume of the Verson’s glands. However, size changes in a saccule cell (indicated by white arrows in **Figure 1C**) was also evident especially in L5 and L6. Verson’s glands are not easily visible under bright light during the feeding stage, but are visible as the gland turn into yellowish color before the molt, especially in L5 and L6 (**Figure 1D**). Verson’s glands undergo programed cell death and disappear by 24 hr after pupation (**Figures 1C, D**).

### 3.2 Genes coding for molting fluid, antimicrobial peptides, and cuticular proteins are highly expressed in Verson’s glands

**Figure 2:**
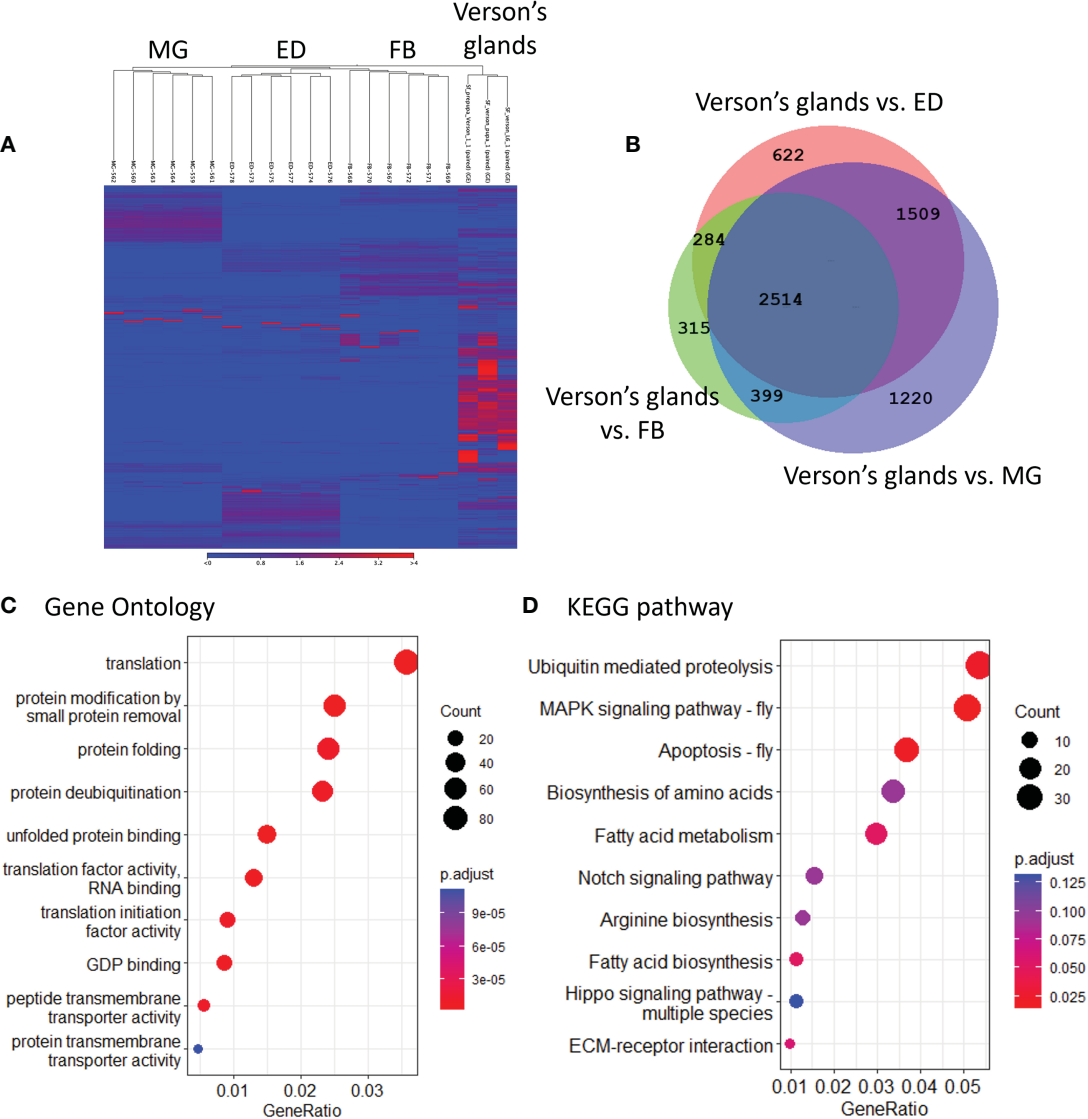
RNA-seq identified genes highly expressed in Verson’s glands. **(A)**, Heatmaps showing the overall comparison between the midgut (MG), epidermis (ED), fat body (FB), and Verson’s glands. The color spectrum, stretching from blue to red, represents TPM values of FAW transcripts calculated from RNA-seq analysis. **(B)**, Venn diagram showing the number of DEGs upregulated in Verson’s glands compared to the midgut, epidermis, and fat body. Enriched Gene Ontology **(C)** and KEGG pathway **(D)** terms in 2,514 DEGs significantly expressed in Verson’s glands.

**Figure 3:**
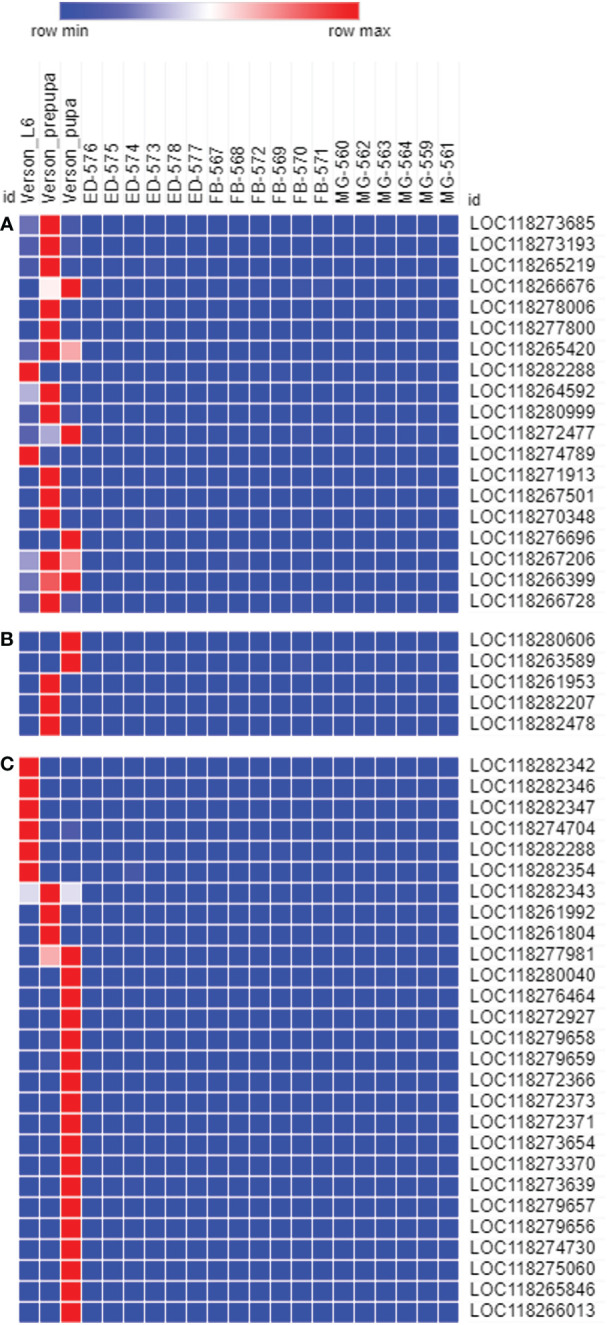
The genes expressed in Verson’s glands. Molting fluid protein **(A)**, antimicrobial peptide **(B)**, and cuticular protein **(C)** coding genes are highly expressed in Verson’s glands. Gene ID descriptions are included in **Table S6**.

To determine the physiological functions of Verson’s glands, we identified genes highly expressed in Verson’s glands using RNA-seq. RNA isolated from Verson’s glands from three different stages (L6 last instar larvae, prepupae, and pupae) were sequenced. Differential gene expression analysis was performed comparing the expression of genes in Verson’s glands with those in the midgut, epidermis, and fat body (**Figure 2A**). 2514 DEGs were identified in Verson’s glands that are expressed at higher levels compared to their expression in the midgut, epidermis, and fat body (**Figure 2B**; **Table S2**). Enriched Gene Ontology terms for the identified DEGs included protein translation, folding, and modification (**Figure 2C**). Enriched KEGG pathway terms included several metabolic biosynthesis pathways and Hippo signaling pathways (**Figure 2D**). Identified DEGs included genes that code for proteins found in molting fluid (19) (**Figure 3A**), antimicrobial peptides (**Figure 3B**), and cuticular protein (**Figure 3C**). There were 52 DEGs that were downregulated in Verson’s glands compared to their expression in the midgut, epidermis, and fat body (**Figure S1A** and **Table S3**). Enriched Gene Ontology terms for the downregulated DEGs included fatty acid metabolic process (**Figure S1B**). Enriched KEGG pathway terms included fatty acid degradation (**Figure S1C**).

### 3.3 Genes coding for hemolymph proteins are highly expressed in Verson’s glands

Previous reports (11, 12) showed that Verson’s glands secrete protein into the hemolymph. We checked whether FAW Verson’s glands indeed contribute to hemolymph proteins. We first used the list of hemolymph proteins identified in the silkworm, *Bombyx mori* (20) to identify FAW homologs. Six hundred and seven genes coding for hemolymph proteins were identified (**Table S4**). Interestingly, 144 genes (24%) are identified as highly expressed in Verson’s glands (**Figure S2A**; **Table S5**). Enriched Gene Ontology terms for 144 genes coding for hemolymph protein highly expressed in Verson’s glands, include vesicles and chitin metabolic processes (**Figure S2B**). Enriched KEGG pathway terms included carbon metabolism and ECM-receptor interaction (**Figure S2C**).

### 3.4 Verson’s glands growth inhibition by RNAi

**Figure 4:**
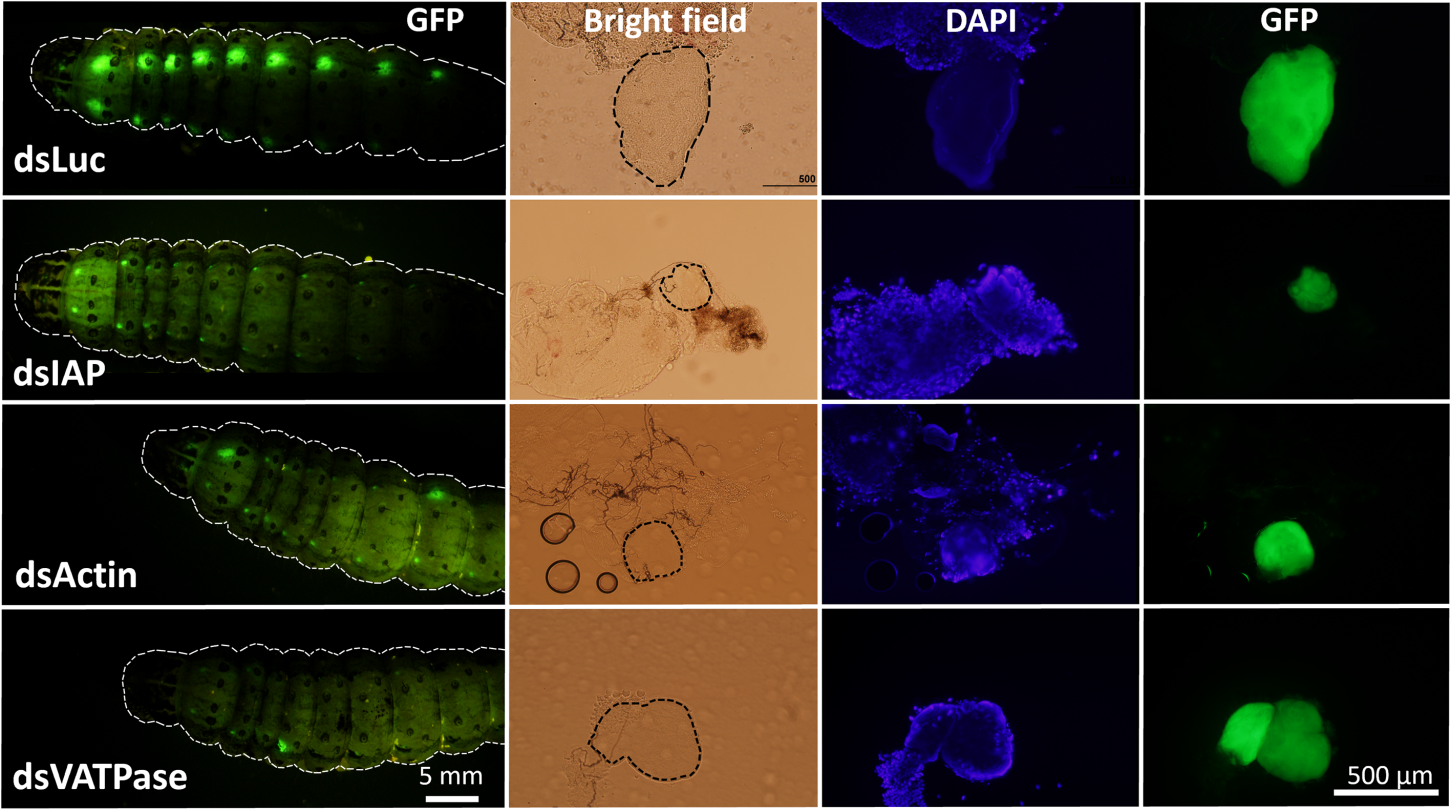
Inhibition of growth and survival of Verson’s glands by RNAi. Eight µg dsRNA (dsLuc, dsIAP, dsActin and dsVATPase) was injected into day 0 last instar FAW-SID1 larva. Five days after injection, when they became prepupa, Verson’s glands were dissected, fixed in 4% paraformaldehyde, and mounted on a glass slide with a mounting medium containing DAPI. Verson’s glands size is indicated by dotted black lines in bright field image.

In our previous study, we observed that RNAi efficiency is highly improved in Verson’s glands of FAW-SID1 strain (13). We knocked down some essential genes, including the IAP, Actin, and VATPase (21–23). Knockdown of these genes significantly influenced the size of Verson’s glands (**Figure S3**), specifically the secretory cell that makes up most of the volume of the gland (**Figure 4**). Although the growth and survival of Verson’s glands were significantly affected, the dsRNA-treated larva developed into pupae and adults (**Figure S4**). There was also no significant mortality observed in the treated larva compared to dsLuc and dsGFP control (**Figure S5**). dsGFP is frequently used as a control in FAW experiments and it does not affect larval development and survival (13, 23–25). Therefore, the basal mortality observed in the assay maybe due to damage from the injection injury.

## 4 Discussion

Molting fluids help recycle cuticular proteins and chitin from the old cuticle (26) and protect the pharate insects with soft cuticles against microorganisms (19). Verson’s glands are believed to contribute to molting fluid secretion (4, 6, 27). Indeed, genes that code for molting fluid proteins, including chitinase, peptidase, and phenoloxidase are highly expressed in Verson’s glands (**Figure 3**; **Table S6**). Out of 93 genes coding for FAW orthologs of molting fluid proteins identified in *B. mori* (**Table S7**), 19 genes are (20%) highly expressed in Verson’s glands (**Figure 3A**; **Table S6**). Molting fluid is presumed to be produced by both epidermis and dermal glands (19, 26), so Verson’s glands may not be the exclusive source of the molting fluid in FAW. Further research is needed to check whether the protein content of the secreted molting fluid is different between Verson’s glands and other molting fluid-secreting sources.

During ecdysis, the old cuticle is shed, revealing a newly formed soft cuticle. This newly molted insect is subjected to infection risk, and it is important for insects to manage this risk during ecdysis. Proteomic analysis of silkworm molting fluid revealed that 9% of the total identified secretory protein accounted for immune-related proteins (28), which indicates considerable investment for protection during molting. Indeed, molting fluid from *B. mori* inhibited the growth of bacteria *in vitro* (19). Verson’s glands are also reported to have an immune function. Nardi et al., identified novel proteins that are related to the immune response in Verson’s glands (11). We identified several antimicrobial peptides, including gallerimycin and inducible metalloproteinase inhibitor proteins highly expressed in Verson’s glands of FAW (**Figure 3B**; **Table S6**). Hippo signaling pathway was enriched in Verson’s glands (**Figure 2D**). Hippo signaling pathway is controlled by Toll signaling pathway to regulate antimicrobial peptide expression (29). Twenty-seven genes coding for cuticular proteins are highly expressed in Verson’s glands. Cuticular protein genes are typically expressed in the epidermis (30). Further studies with different developmental stages are needed to determine whether the expression of cuticular protein-coding genes in Verson’s glands have specific functions or redundancy with those made in the epidermis. However, it looks clear that Verson’s glands contribute to cuticular protein production.

Secretions from insect epidermal glands use duct cells to go through the outer cuticle and reach extracellular space (31). Verson’s glands are composed of three cells, a secretory cell, a saccule cell, and a duct cell. There is a channel connecting the duct cell and the saccule cell. However, there is no evidence of connecting channel between the duct cell and the secretory cell. (11). Ultrastructural analysis showed that during molt, vacuoles in secretory cells invaginate in basal lamina and discharge materials into the hemolymph (12). These observations suggest that the secretory cells of Verson’s glands secrete proteins into the hemolymph directly. Indeed, we identified 144 hemolymph protein genes that are highly expressed in Verson’s glands (**Figure S2A**). These proteins seem to function in multiple metabolic pathways, including chitin catabolism (**Figure S2B**). The fat body is the major source of hemolymph protein production (32). Our study together with previous reports suggest that Verson’s glands may also be one of the important sources of hemolymph proteins especially during molting. We observed that similar to secretory cells, saccule cells also grow during feeding period and reduce in size after molting (**Figure 1C**). Proteins produced in saccule cells may use the channel connected with duct cell to discharge them outside the cuticle. Taken together, it appears that macromolecules produced from Verson’s glands can be secreted to both outside and in to hemolymph from a saccule cell and a secretory cell respectively. Further studies are needed to confirm this hypothesis.

We injected dsRNA targeting housekeeping genes required for cell survival and observed a significant reduction of cell size in Verson’s glands (**Figure 4**). We observed a slight decrease in GFP fluorescence in the Verson’s glands of the treated larva as well (**Figures 4**, **S3**). It is likely that knockdown of housekeeping genes affected the cell viability and affected overall protein production including GFP protein. However, this treatment didn’t affect overall larval development (**Figure S4**) and didn't cause significant mortality (**Figure S5**). This result is consistent with our previous report where nearly 80% of injected FAW-SID1 in both control and dsIAP treatment successfully pupated (13). In our previous study, dsGFP injection in FAW-SID1 larva only decreased GFP fluorescence level in Verson’s glands and not in other tissues. dsGFP injection resulted in 93.3% knockdown of GFP gene expression in Verson’s glands. There were moderate but significant knockdown in Malpighian tubules and testis, but no knockdown was observed in rest of the tissues (epidermis, fat body, midgut, ovary, and salivary glands) (13). Although we didn’t measure knockdown level of the target genes (IAP, Actin, VATPase) in different tissues in this study, it is most likely that our treatment mainly affected Verson’s glands and not in other tissues like what we observed in our previous study. These results suggest that Verson’s glands may not be required for pupal ecdysis. It is possible that although the dsRNA treatment significantly impairs the growth of Verson’s glands, there is still some protein secretion. Another possibility is that the larvae without Verson’s glands may only be affected when they are challenged with microorganisms that cause diseases. Further studies are needed to understand the impact of Verson’s glands on larval development in different physiological contexts.

This study reports morphological characteristics of Verson’s glands in different developmental stages of FAW, and their transcriptomic analysis. We identified highly expressed genes in Verson’s glands to verify previous observations in Verson’s glands physiology. Our results showed that Verson’s glands might contribute to cuticular protein production, molting fluid, hemolymph protein production, and immune response. Our study provides valuable tools for further research in identifying the physiological significance of Verson’s glands in lepidopteran insects.

## Data availability statement

The datasets presented in this study can be found in online repositories. The names of the repository/repositories and accession number(s) can be found in the article/**Supplementary Material**.

## Author contributions

JK and SP designed research; JK and XC performed research; JK and SP analyzed data; JK and SP wrote the paper. All authors contributed to the article and approved the submitted version.
