# Supplementary material for: Gene expression in Verson’s glands of the fall armyworm suggests their role in molting and immunity
Source: Front Insect Sci. 2023 Feb 10;3:1124278. doi: 10.3389/finsc.2023.1124278 (PMC10926397; doi:10.3389/finsc.2023.1124278)
Supplement: Supplementary file 1 [file DataSheet_1.pdf]

Supplementary information

Gene expression in Verson's glands of the fall armyworm suggests their role in molting and immunity

Jinmo Koo [a], Xien Chen [ab], and Subba Reddy Palli [a*]

[a] Department of Entomology, College of Agriculture, University of Kentucky, Lexington, KY 40546, USA
[b] Current address, State Key Laboratory of Crop Stress Biology for Arid Areas, College of Plant Protection, Northwest A&F University, Xianyang 712100, China

*Corresponding author: rpalli@uky.edu

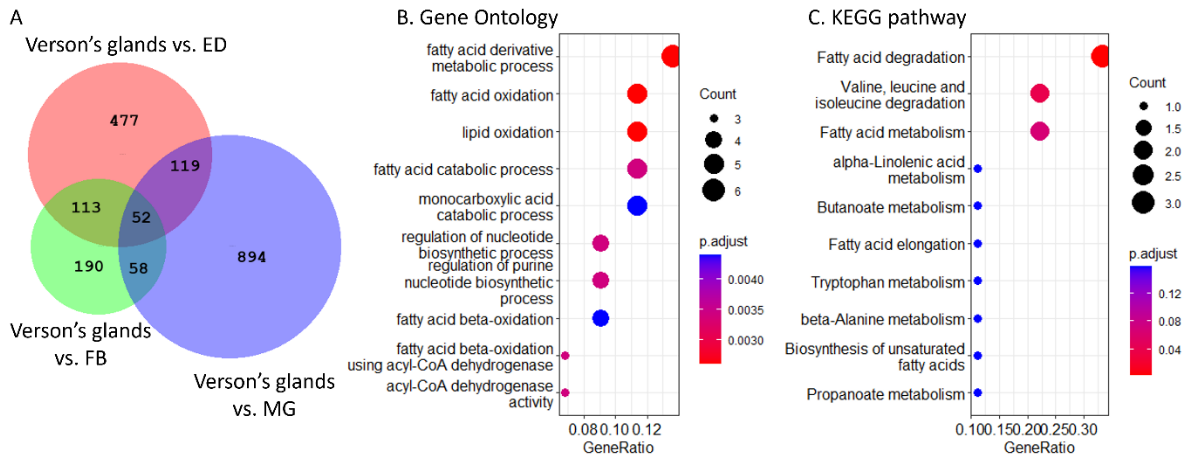

Figure S1. Down regulated genes in Verson's glands. [A], Venn diagram showing the number of DEGs down regulated in Verson's glands compared to the epidermis (ED), fat body (FB), and midgut (MG). Enriched Gene Ontology [B] and KEGG pathway [C] terms in 52 DEGs significantly down regulated in Verson's glands.

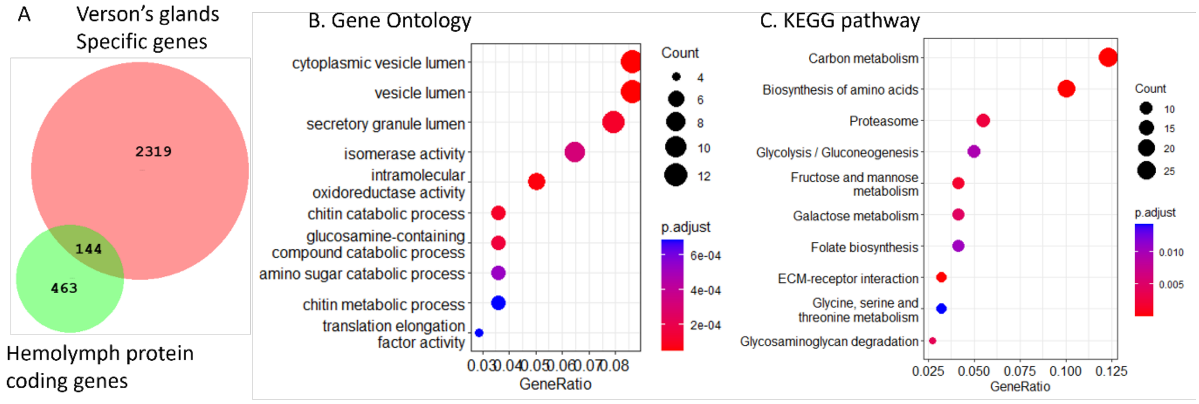

Figure S2. Hemolymph protein genes highly expressed in Verson's glands. [A], FAW orthologs of silkworm genes coding for hemolymph proteins expressed in Verson's glands were identified. Enriched Gene Ontology [C] and KEGG pathway [D] terms in 144 hemolymph protein genes expressed in Verson's glands.

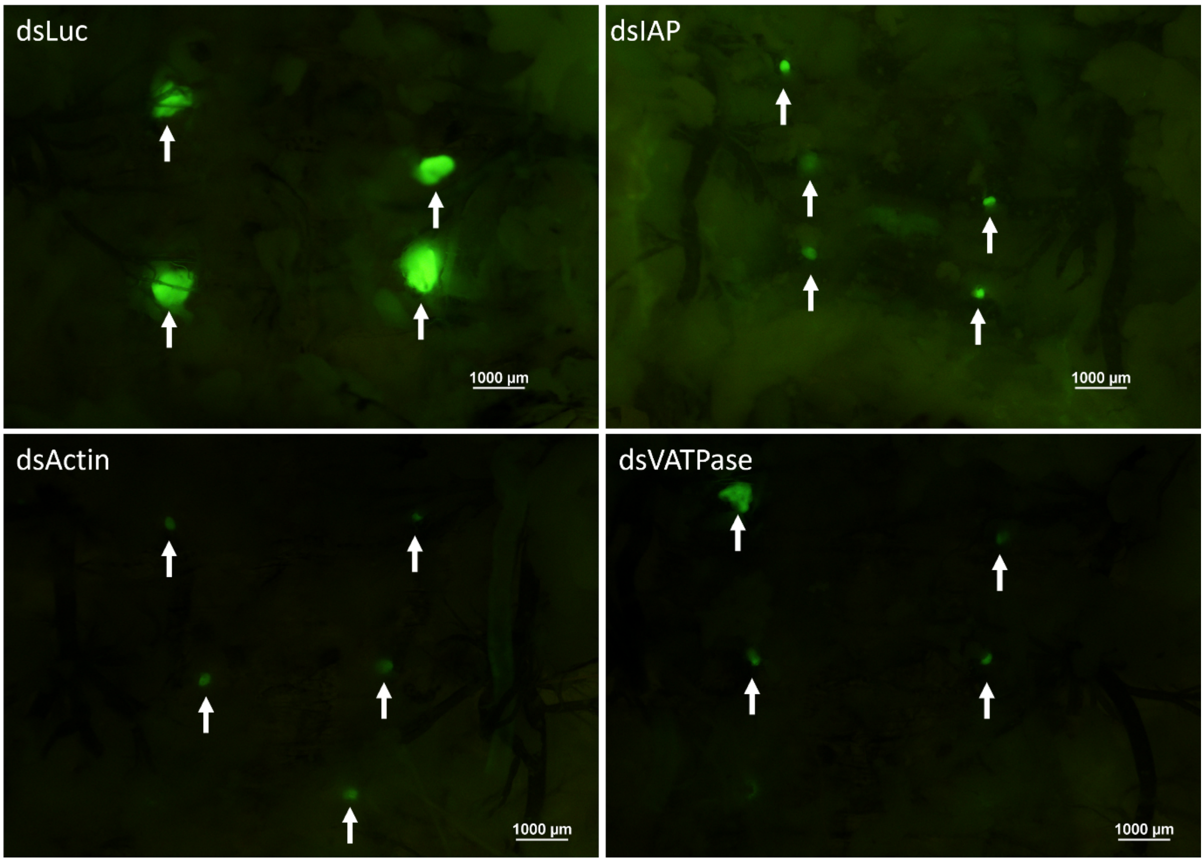

Figure S3. Verson's glands affected by RNAi. Eight µg dsRNA (dsLuc, dsIAP, dsActin and dsVATPase) was injected into day 0 last instar FAW-SID1 larva. Five days after injection, when they became prepupa, they were cut open and Verson's glands were photographed under fluorescent light.

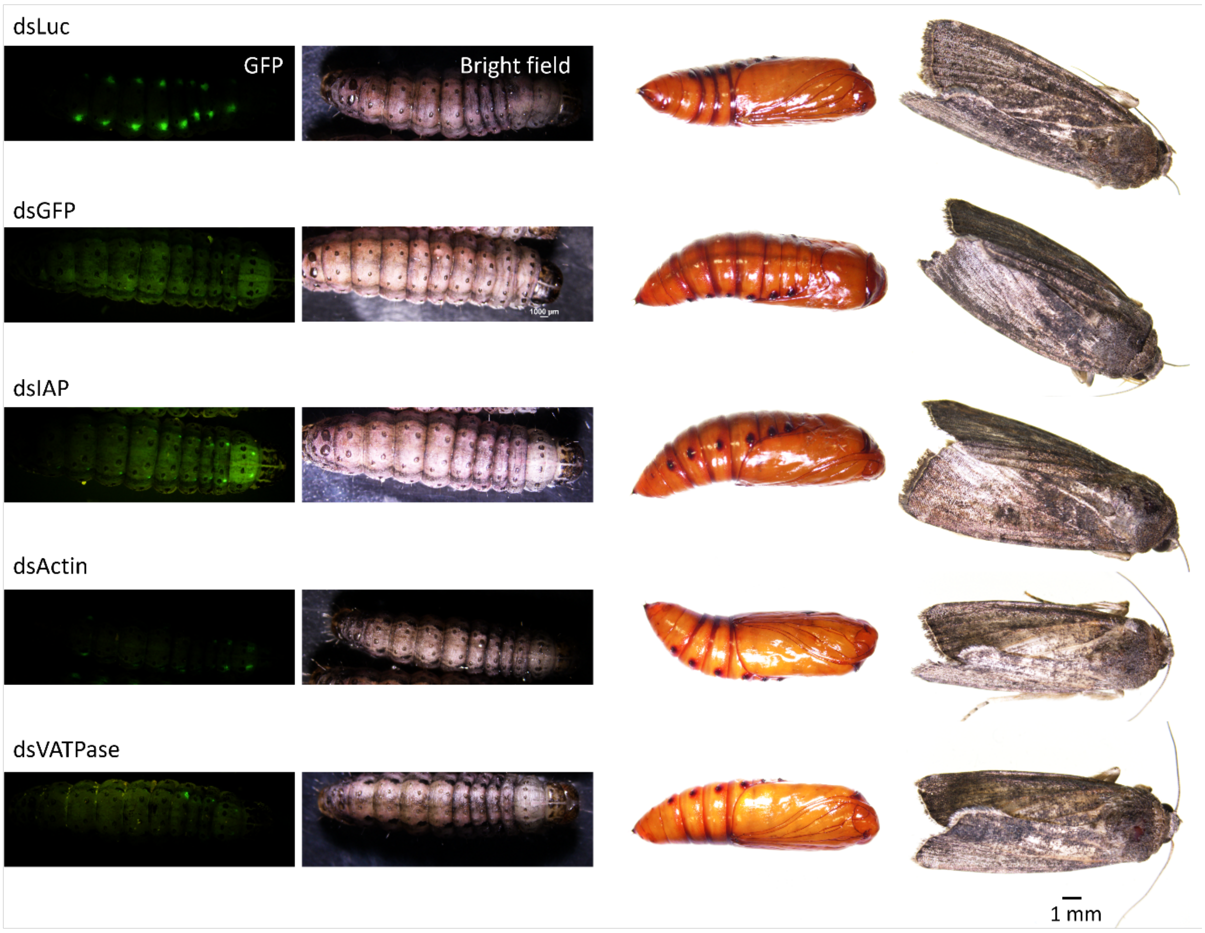

Figure S4. Inhibition of growth in Verson's glands didn't affect FAW development. Eight μg dsRNA (dsLuc, dsGFP, dsIAP, dsActin and dsVATPase) was injected into day 0 last instar FAW larva. Photographs of representative individuals in prepupa, pupa, and adult stages were taken.

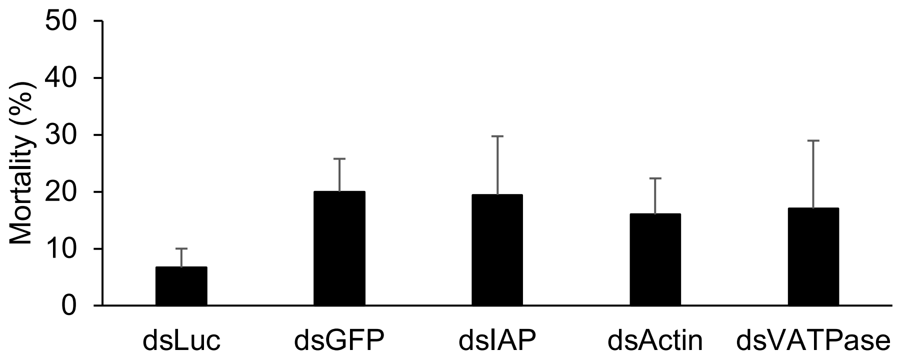

Figure S5. Inhibition of growth in Verson's glands did not induce significant mortality. Eight μg dsRNA (dsLuc, dsGFP, dsIAP, dsActin and dsVATPase) was injected into each day 0 last instar FAW larva. Mortality was recorded until adult emergence. Mean + S.E (n = 9~12) of three replicates are shown.
